# A cognitive evaluation and equity-based perspective of pay for performance on job performance: A meta-analysis and path model

**DOI:** 10.3389/fpsyg.2022.1039375

**Published:** 2023-01-20

**Authors:** Yuyao Chen, Zhengtang Zhang, Jinfan Zhou, Chuwei Liu, Xia Zhang, Ting Yu

**Affiliations:** ^1^Business School, Nanjing University, Nanjing, China; ^2^School of Management, Wuhan University of Technology, Wuhan, China

**Keywords:** pay for performance, job performance, cognitive evaluation, equity, meta-analysis

## Abstract

Pay for performance, as one of the most important means of motivating employees, has attracted the attention of many scholars and managers. However, controversy has continued regarding whether it promotes or undermines job performance. Drawing on a meta-analysis of 108 independent samples (*N* = 71,438) from 100 articles, we found that pay for performance was positively related to job performance. That pay for performance had a more substantial positive effect on task performance than contextual performance in workplace settings. From the cognitive evaluation perspective, we found that pay for performance enhanced employees' task performance and contextual performance by enhancing intrinsic motivation and weakened task performance and contextual performance by increasing employee pressure. From the equity perspective, our results indicated that the relationship between pay for performance and task performance was partially mediated by employee perceptions of distributive justice and procedural justice, with distributive justice having a more substantial mediating effect than procedural justice. However, the relationship between pay for performance and contextual performance was only partially mediated by procedural justice. Further tests of moderating effects indicated that the varying impacts of pay for performance are contingent on measures of pay for performance and national culture. The findings contributed to understanding the complex mechanisms and boundary conditions of pay-for-performance's effects on job performance, which provided insights for organizations to maximize its positive effects.

## Introduction

An important concern for employers is how to encourage employees to show high job performance in organizational practice. Pay for performance (PFP) refers to any pay program for employees in which some or all of their pay depends on their individual or organizational performance (e.g., merit pay, individual and/or team bonus pay, profits-sharing and stock plans) (Gerhart and Fang, 2015; Nyberg et al., 2016; Kong et al., 2022; Park and Conroy, 2022). Moreover, it is seen as one of the essential means for employers to motivate their employees and has received much attention from researchers and employers (Gerhart et al., 2009; Gerhart and Fang, 2014). However, for nearly half a century, scholars have been conflicted about whether and how PFP enhances or undermines employee job performance.

Studies based on the economic and initial psychological perspectives all emphasized the incentive effects of PFP on job performance. The economic perspective suggests that individuals react rationally and self-interestedly in the face of external incentives (Milgrom and Roberts, 1992). Since PFP enables extra effort to result in incremental payoffs, employees improve their performance to maximize their pay, as suggested by the incentive intensity principle (Jensen and Meckling, 1976). The early psychological perspective, such as expectancy theory (Vroom, 1964), states that PFP has an incentive effect on individual performance, significantly when individuals recognize the value of pay, are convinced that high performance will result in high pay, and believe that they can change their performance by putting in the effort. Several empirical studies support the positive effects of PFP on job performance (e.g., Chang and Hahn, 2006; Nyberg et al., 2016; Maltarich et al., 2017; Kong et al., 2022).

In contrast, later cognitive psychological perspectives, represented by Deci and Ryan (1985), challenged the incentive effects of PFP. For example, cognitive evaluation theory and self-determination theory point out that PFP undermines employees' intrinsic motivation and thus reduces their work efforts. Although this idea triggered a major crisis regarding the incentive effects of PFP, it also led scholars to shift from simply considering “PFP-performance” to focusing on the psychological processes that link PFP to performance.

Prior meta-analyses have examined the relationship between PFP and performance (e.g., Jenkins et al., 1998; Weibel et al., 2010; Garbers and Konradt, 2014; Kim et al., 2022), but all were conducted based on the findings of experimental studies. Experimental studies are overly simplistic in both the operationalization of PFP and the assessment of performance. For example, PFP was manipulated using either reward or no reward (Allscheid and Cellar, 1996; Hobson et al., 2021), and performance was measured in terms of the quantity or quality of completion of specified tasks (Whitehill and McDonald, 1993; Cadsby et al., 2007). Such simplified experimental manipulations may reflect only part of the relationship between PFP and performance. First, in an actual workplace, the opposite of PFP may not be “zero,” but rather “fixed pay,” or PFP may be more finely represented as different intensities of PFP. Second, in actual workplace requirements, job performance involves not only aspects of completing in-role tasks, but also some extra-role aspects of work, such as volunteering overtime, and organizational citizenship behaviors, which are referred to as contextual performance (Borman and Motowidlo, 1993; Motowidlo and Van Scotter, 1994; Rich et al., 2010). Also, PFP in the workplace means not only getting more if you do more but also losing more if you get less. In other words, PFP also means risk and uncertainty (Tosi Jr and Gomez-Mejia, 1989). These issues cannot be fully considered in a meta-analysis based on experimental studies. Therefore, the first question we want to answer through our meta-analysis is: Does PFP promote or weaken employee job performance in real work settings, and to what extent does it have an effect? Does it have a differential effect on task performance and contextual performance?

Although multiple studies have explored the relationship between PFP and performance, the underlying mechanisms through which PFP affects performance remain incomplete. As a representative of psychological perspectives, cognitive evaluation theory (Deci and Ryan, 1985) has been devoted to unlocking the psychological mechanisms between PFP and job performance. The growth of PFP literature has also witnessed the rise of cognitive evaluation theory from an emerging lens to perhaps the dominant lens explaining the PFP effects. According to cognitive evaluation theory, PFP has both informational and controlling aspects, so that PFP can exert opposite effects on job performance through informational and controlling mechanisms. Although past meta-analytic studies (e.g., Jenkins et al., 1998; Kim et al., 2022) have mentioned the critical connecting role of cognitive-related concepts such as intrinsic motivation in the relationship between PFP and performance, these discussions are relatively minor in their discourse. More importantly, these meta-analytic articles do not answer the core issues of PFP research using a cognitive evaluation approach. For example, to what extent do informational and controlling mechanisms mediate the effects of PFP on job performance, respectively? Which mechanism explains the PFP-performance relationship more strongly? These are the second question that our meta-analysis seeks to address.

As PFP research has evolved, scholars have gradually moved beyond the assumption that people are rational and self-interested to realize the impact of PFP on employees' irrational behaviors (Merriman and Deckop, 2007; Gläser et al., 2017; He et al., 2021). Employees' perceived fairness or unfairness affects their in-role performance and their trust or commitment to their organizations, which is directly related to whether they will engage in extra-role behaviors that are beneficial to the organization, such as organizational citizenship behaviors (Colquitt et al., 2013). Equity theory (Adams, 1963) may be a starting point to explore the mechanisms by which PFP works on different coping strategies of employees (Garbers and Konradt, 2014). None of the meta-analytic studies included the examinations of PFP on justice. Only one meta-analytic review (Garbers and Konradt, 2014) cited seminal conceptual articles on equity theory (e.g., Honmans, 1961; Adams, 1963). Therefore, the third question we hope to answer through our meta-analytic study is: Does PFP predict perceived distributive justice and procedural justice? To what extent do distributive justice and procedural justice mediate the relationship between PFP and job performance (e.g., task performance and contextual performance)?

The fourth issue of concern in our meta-analysis is whether there are validated boundary conditions that can explain the inconsistent conclusions of the current studies. Our literature review found that existing studies have operationalized PFP in different ways, which can be broadly classified into four categories: perception, proportion, amount, and adoption. “Perception” operationalizes PFP as a subjective perception of the pay-performance link. “Proportion” measures PFP as a percentage of performance-related pay in the total compensation. “Amount” refers to PFP as the amount of the performance-based component of pay. “Adoption” refers to the presence or absence of PFP, which has been included in most previous meta-analyses (e.g., Jenkins et al., 1998; Weibel et al., 2010; Garbers and Konradt, 2014). Different measures tend to reflect only one aspect of PFP, and we believe it is necessary to examine which PFP operationalization produces a more profitable predictive effect on performance and other outcome variables. Furthermore, with the increasing global adoption of PFP, it would be beneficial for realistic enterprise management to examine whether there are differences in the effects of PFP across national cultures. Therefore, it is also necessary to consider the moderating effect of national culture on the relationship between PFP and employee outcomes.

This research aims to provide a more accurate view of how PFP affects job performance in the workplace and explore the mechanisms through which PFP contributes to job performance. To do so, we only included studies conducted in real work settings in meta-analysis and developed the meta-analytic structural equation models based on cognitive evaluation theory and equity theory, respectively. In addition, we examine the moderating effects of PFP operationalization and national culture on the relationship between PFP and employee outcomes to provide new insights for a more comprehensive and accurate understanding of the effects of PFP on employees.

This research makes significant contributions to the literature on PFP. First, our meta-analysis focus on studies conducted in actual work settings has the potential to challenge and extend current thinking about the incentive effect of PFP. Existing meta-analytic reviews (e.g., Jenkins et al., 1998; Weibel et al., 2010; Garbers and Konradt, 2014; Kim et al., 2022) primarily based on highly controlled experiments, with the assumption that PFP works in such controlled conditions as it does in the workplace. However, in real work settings, there is a greater amount of pay, longer hours involved, and a greater need for employees to be paid for their work (Rynes et al., 2005). These indicate that a meta-analysis based on experimental studies may not conclude the actual effect of PFP on employees' performance in the real workplace (Weibel et al., 2010). In response to Rynes et al. (2005)'s call to “move to the field,” our meta-analysis summarizes the effect of PFP on job performance by including only those studies that were conducted in real work settings (as opposed to laboratories). In doing so, we hope to open a new avenue for a more accurate summary of the incentive effect of PFP on job performance in the workplace.

Second, we explore the meditating mechanism to explain how and why PFP effects transfer to employee job performance. We focus on cognitive evaluation mechanisms and justice mechanisms, as cognitive evaluation theory and equity theory are among the few theories that focus on the underlying psychological mechanisms of PFP effects and play a pivotal role in PFP research (Gerhart and Fang, 2015). From the cognitive evaluation perspective, we predict that PFP will affect employees' intrinsic motivation and pressure, which can subsequently influence employees' task performance and contextual performance. From the equity perspective, we predict that PFP will affect employees' perceptions of distributive justice and procedural justice, which can contribute to two job performance types. By examining the mediating mechanisms in our research, we respond to calls for further understanding of “the psychological mechanisms that explain employee reactions to [PFP] plans” and help “identify why [PFP] plans do not always work as intended” (Rynes et al., 2005, p. 573).

Finally, most PFP studies confirm the positive impact of PFP on employee outcomes. However, there remains a notable amount of variability among the empirical findings, suggesting the existence of potential moderating variables that sway observed estimates. Therefore, the third contribution of our research is to explore the moderating effects of national culture and PFP measurement on the relationship between PFP and employee outcomes.

## Hypotheses

### Pay for performance and job performance

Linking employees' performance and their pay is the core of PFP. This incentive motivates employees to work harder to maximize their pay (Milgrom and Roberts, 1992). Agency theory (Jensen and Meckling, 1976) states that the principal could incentivize agents by controlling their financial incentives. The principal (in the real work settings, is the employer) aligns the agents' (employees, correspondingly) interest with its own by linking agents' pay with their performance, which motivates agents to maximize their performance to get what they want (money). Thus, rather than being an incentive theory, agency theory is more of a control theory to help the principle to monitor agents' behaviors. In expectancy theory, an individual's effort is the product of the expectation (the probability that an individual's effort can result in desired performance), instrumentality (the perceived probability that desired performance will lead to expected compensation), and valence (the value that the individual placed on the reward as compared to other outcomes, such as stress, less leisure time) (Vroom, 1964). Therefore, PFP strongly affects individual performance if the link between effort, performance, and rewards is obvious. In equity theory (Adams, 1963), employees determine whether they are being treated fairly by comparing their pay with others and with themselves, respectively. Equity does not exactly mean equality, while equity is a balanced perception of input and outcome. When employees perceive equity, they are motivated to invest more effort, resulting in high job performance. As a fairness-oriented compensation system, PFP reflects the value of “more work, more pay” and increases employees' perception of justice, thus helping to improve employee job performance. In cognitive evaluation theory, the effect of PFP seems to be variable. PFP affects employee performance in two ways: control and information (Deci and Ryan, 1985). When PFP is interpreted as an informative system, it can motivate employees to maximize their performance (Fisher, 1978). Conversely, when PFP is perceived as a controlling system, employees perceive themselves under external threat and pressure, which drives burnout and reduces work effort (Yeh et al., 2009; Kuvaas et al., 2016).

Although the findings on the relationship between PFP and job performance are inconsistent, most studies support the incentive effect of PFP on job performance. For instance, In Chien et al. (2010)'s study, when R&D professionals were rewarded according to their job performance, they tended to focus on the job performance, which verified the motivational effect of PFP. Based on the 3-year longitudinal data from the health care industry, Maltarich et al. (2017) also found that PFP positively affects individual performance. Thus, we predicate a strongly positive relationship between PFP and job performance.

Previous meta-analyses on PFP and job performance have classified performance as quantitative performance and qualitative performance (Garbers and Konradt, 2014; Kim et al., 2022), or directly and integrally as job performance (Jenkins et al., 1998; Weibel et al., 2010). However, performance is a multidimensional concept, and each dimension expresses different aspects of performance, so it is necessary to distinguish the specific effects of PFP on different dimensions of performance. Researchers acknowledge that task performance and contextual performance are two distinct aspects of job performance, and each has unique contributions to overall job performance (Borman and Motowidlo, 1993; Motowidlo and Van Scotter, 1994; Van Scotter et al., 2000; Rich et al., 2010). Task performance refers to those activities that are more closely related to the content of the job and are formally required by the organization to be completed. On the contrary, contextual performance refers to those voluntary activities that are beneficial to the organization but are not required by it. In agency theory (Jensen and Meckling, 1976), PFP will likely motivate employees to focus more on their in-role behaviors, such as task performance (Jensen and Meckling, 1976; Milgrom and Roberts, 1992). A possible by-product is that employees may reduce un-rewarded behaviors, such as contextual performance (Deckop et al., 1999). Several studies show the different effects of PFP on both dimensions of job performance (Deckop et al., 1999; Du and Choi, 2009; Chien et al., 2010; Auh and Menguc, 2013; He et al., 2021). In line with previous studies, we expect PFP has unique effects on task performance and contextual performance.

*Hypothesis 1a: PFP is positively related to job performance*.
*Hypothesis 1b: PFP has a stronger association with in-role performance (e.g., task performance) than with extra-role performance (e.g., contextual performance)*.

### The emerging importance of cognitive evaluation theorizing in the PFP literature

Although the PFP literature has focused a great deal of theoretical attention on whether and why PFP produces positive effects on employee outcomes, such as job performance, examination of how PFP works, especially how it works on job performance, is an ongoing theme. Gerhart and Milkovich (1992) noted that there needs to be more psychological research on the relationship between PFP and outcomes, because only a better understanding of the psychological mechanisms by which PFP drives employees can explain “why [PFP] plans do not always work as intended” (Rynes et al., 2005, p. 573). Deci and Ryan (1985) proposed the cognitive evaluation theory based on the liberal and individualistic ideas of the Romantic philosophical view and pointed out that intrinsic motivation is the original driving power that drives people to perform behavioral activities. Individuals will cognitively evaluate the external environment (e.g., PFP) to determine whether it can support their claim to freedom. An informative environment or thing enhances an individual's intrinsic motivation, whereas a controlling reward makes the individual feel pressured and tends to undermine intrinsic motivation (Ryan, 1982; Ryan et al., 1983).

Cognitive evaluation theory has led scholars, in a real sense, to focus on the psychological processes between pay and outcomes. As one of the few theories that focus on the underlying psychological mechanisms of PFP, cognitive evaluation theory plays an essential role in PFP research (Gerhart and Fang, 2015). Rynes et al. (2005) labeled cognitive evaluation theory as one of the most influential psychological theories explaining the effectiveness of PFP in the workplace. Therefore, we may believe that integrating cognitive evaluation theory and PFP is very useful in explaining why PFP may enhance or reduce employees' task performance and contextual performance.

### A cognitive evaluation perspective: The mediating role of intrinsic motivation and pressure

Just like two sides of a coin, PFP can be both a stressor and a motivator for employees. Cognitive evaluation theory (Deci and Ryan, 1980, 1985) suggests that PFP has both informational and controlling aspects. For the controlling aspect, the PFP tends to be experienced as controlling if the PFP signifies that employees must meet their own performance goals, so they can earn the pay raise, in other words, forcing employees to do something (Ryan et al., 1983). When employees perceive PFP as controlling, they will feel pressure to meet performance goals and experience stress reactions such as anxiety, insomnia, and even depression. A survey of nearly 300,000 employees in Danish companies showed that employees were 5.7% more likely to use anxiolytics or antidepressants in companies with PFP (Dahl and Pierce, 2020). Some studies also supported the positive relationship between PFP and pressure (Fitzpatrick, 2008; Habel et al., 2021). As a result, employees will try to reduce pressure in a variety of ways, such as decreasing work efforts, declining performance (Yitzhak et al., 2013), engaging in counterproductive behaviors (Carpenter and Berry, 2017), or leaving the company (Yitzhak et al., 2013; Dahl and Pierce, 2020). Therefore, we predicted that PFP would increase employees' pressure, and pressure would mediate the relationship between PFP and job performance.

For the informational aspect, the PFP tends to be experienced as information if the PFP signifies to employees that they are capable of doing their jobs and that receiving pay raises means they are performing well, in other words, increasing employees' perceptions of competence. When employees perceive PFP as information, their interest in the work itself increases, which is called “intrinsic motivation” in motivation research. Several studies confirmed that PFP enhances intrinsic motivation (Cabanas et al., 2020). Hackman and Oldham (1975) suggested that intrinsic motivation significantly influences employees' behaviors and attitudes. Employees with high intrinsic motivation tend to care more about their work and actively seek better ways to address challenges in their work. Also, previous studies confirmed that intrinsic motivation mediates the relationship between PFP and its outcomes. For instance, creativity researchers found that intrinsic motivation mediated the relationship between PFP and creativity (Zhang et al., 2015a, 2021). Thus, we suspect that PFP would positively relate to employee intrinsic motivation, and intrinsic motivation will mediate PFP and job performance.

*Hypotheses 2a– 2b: PFP is positively related to (a) intrinsic motivation and (b) pressure*.
*Hypothesis 3: The relationship between PFP and job performance (e.g., task performance, and contextual performance) is mediated by intrinsic motivation and pressure*.

### Another explanation: The non-negligible importance of justice

Although cognitive appraisal theory provides a nuanced framework for explaining the PFP effect in terms of psychological mechanisms, this explanation is relatively emotional because it is the employees' cognitive appraisal of PFP about their own needs for competence and autonomy. In contrast, equity theory (Adams, 1963) offers an alternative perspective to explain the PFP effect with a relatively rational psychological-cognitive paradigm. According to equity theory, people in social exchange relationships (in compensation matters, economic exchange only) believe that pay should be given based on how much each member contributes (Adams, 1963, 1965; Walster et al., 1973). Employees will compare their input-output ratio with the reference and consider it fair only if they are equal (Cowherd and Levine, 1992). Otherwise, they will alter their actual input, such as reducing work efforts, decreasing organizational citizenship behavior, or leaving the organizations (e.g., Colquitt et al., 2002, 2013; Chien et al., 2010).

By linking employees' income with their job performance, PFP is completely consistent with the principle of “everyone should be paid fairly” reflected in the equity theory (Du, 2009). Therefore, performance pay is considered a fair-oriented compensation system (Du and Choi, 2009). More scholars have paid attention to the important explanatory role of equity theory for the PFP effect in recent years. For example, Chang and Hahn (2006) investigated the impact of PFP on employees' perceptions of distributive justice in a Korean sample. Uriesi (2017) focused on both distributive justice and procedural justice in explaining the PFP effects. Although equity theory has a place in the PFP literature, it is not at the core. In other words, the PFP and equity literature integration have been slower than expected. Therefore, it is necessary to integrate the two within a framework through a quantitative review to provide an alternative perspective for explaining the psychological mechanisms of the PFP effect.

### An equity perspective: The mediating role of justice

Researchers recognize that distributive justice and procedural justice are two distinctive aspects of organizational justice. In the PFP situation, distributive justice is defined as the perceived fairness of pay outcomes, especially focusing on the compensation employees received. Procedural justice is defined as the perceived fairness of the payment process, especially focusing on the transparency of compensation allocation and the opportunities to voice (Colquitt et al., 2013; Zhang et al., 2015b).

Previous research indicated that compensation practice is one of the most important factors affecting employees' justice perception. In actuality, higher-paid employees are more likely to believe they are being treated fairly, and the study of Newman and Milkovich (1990) supports this idea. PFP, as a compensation practice, enables high performers to be paid more, thus enhancing employees' perception of justice. In addition, because PFP reflects the values of “more work, more pay,” it increases employees' sense of control over their pay (self -determination), which is consistent with the logic of control in equity (Leventhal, 1980). According to equity theory (Adams, 1963), the sense of unfairness comes from imbalance, including one's input-output imbalance and self-other imbalance. PFP enables employees to receive highly correlated pay with their contribution (e.g., in-role effort), reducing the sense of unfairness caused by imbalanced comparison results. Researchers have recognized that distributing pay based on employee performance will promote employees' perceptions of distributive justice and procedural justice (Campbell et al., 1998; St-Onge, 2000; Du and Choi, 2009; Chien et al., 2010; Zhang et al., 2015b). In line with previous studies, we stated that PFP positively affects these two constructs.

Consistent with the logic that PFP affects justice, PFP should have a positive relationship with pay satisfaction. When employees perceive performance as helpful in achieving valuable outcomes, such as a pay raise, their pay satisfaction increases (Heneman et al., 1988; Schaubroeck et al., 2008; Shi et al., 2016). Additionally, when employees' input-output ratio is equal to that of a referent, they will be satisfied with their pay. PFP makes it easier to reach an equal ratio, thus enhancing pay satisfaction. Green and Heywood (2008) showed that the PFP increased overall employee satisfaction, pay satisfaction, job satisfaction, and job hours satisfaction. Evidence for a cross-culture sample in the US and Hong Kong also supports this effect. Thus, we hypothesize that:

*Hypotheses 4a*−*4c: PFP is positively related to (a) distributive justice, (b) procedural justice, and (c) pay satisfaction*.

Building on previous employee justice perception studies, we explored the mediating mechanism by which PFP affects job performance. As discussed above, PFP enhances employees' perceptions of justice. Also, several studies showed that a lack of justice would lead to unfavorable outcomes, such as poor task performance, less organizational citizenship behaviors, and some negative attitudes in the workplace (Chien et al., 2010; Colquitt et al., 2013; Hietapakka et al., 2013). Especially, there is a significant difference in the effects between the two types of justice: employees' perceptions of procedural justice had a more significant effect on organizational citizenship behavior and organizational commitment than perceptions of distributive justice, while employees' task performance and job satisfaction are more directly influenced by distributive justice (Viswesvaran and Ones, 2002; Colquitt et al., 2013). Based on these findings, we suspected that increasing the intensity of PFP would improve employees' perceptions of distributive justice and procedural justice, which would then exhibit unique effects on task performance and contextual performance. Additionally, Williams et al. (2006)'s meta-analysis found a strong relationship between pay satisfaction and justice perceptions, so we expect justice to mediate the PFP-performance relationship after controlling for pay satisfaction.

*Hypothesis 5: The relationship between PFP and job performance (e.g., task performance, and contextual performance) is mediated by distributive justice and procedural justice*.

### PFP operationalization as a moderator of the PFP-outcomes relationships

Inconsistent findings between each PFP-outcomes relationship may result from how PFP is measured. Extent research on the operationalization of PFP can be categorized into two camps: subjective and objective measurements. For subjective measurement, PFP was operationalized as a “perception,” referring to the association between performance and pay perceived by the employees. A wildly used scale was Deckop et al. (1999)'s 3-item scale, which asked employees to evaluate the degree of performance-pay link, subjectively. The sample item is “Increased productivity means higher pay for employees.” For the objective measurement, PFP was operationalized as a “proportion,” referring to the proportion of performance-based pay in one's total pay. This proportion can be reported by employees or calculated from organizational archival data. Furthermore, PFP can also be measured by the total amount of performance-based pay or the adoption of a PFP system.

Du and Choi (2009) pointed out that the objective measurement of PFP reduced the level of ambiguity, and is closer to the actual PFP than the subjective measurement. However, objective measurements of PFP do not necessarily capture the more realistic impact of PFP. Cadsby et al. (2007) suggested that perceived PFP by employees may better reflect the effects of PFP on employees' attitudes and behaviors than actual PFP.

Scholars have long recognized that actual PFP (“PFP proportion,” in this study) and perceived PFP (“PFP perception,” in this study) have different effects on employee outcomes, although these are two highly correlated concepts (St-Onge, 2000). We predicted that the PFP operationalized as perception is more strongly related to outcomes variables than is PFP measured by other objective measurements (e.g., proportion, amount, and adaptation). First, the objective measurement of PFP assumes that employees can fully and equally feel the PFP implemented by the organization. In fact, organizations may vary in the ability to convey the PFP they implemented, so the PFP perceived by employees is not the same as the PFP implemented by the organization (Deckop et al., 1999). Second, employees with different risk preferences and reward sensitivities will have different PFP perceptions of the same PFP, making PFP produce different effects (Fulmer and Shaw, 2018). People believe more in what they feel, and thus perceived PFP will have a greater impact on the outcome variable than actual PFP. Third, PFP creates a competitive atmosphere in which people compare themselves to each other. Perhaps this feeling of comparison has a stronger impact on employees than the actual PFP. Thus, we hypothesize that:

*Hypotheses 6a-6g: PFP operationalized as a perception is more strongly related to (a) task performance, (b) contextual performance, (c) distributive justice, (d) procedural justice, (e) pay satisfaction, (f) intrinsic motivation, and (g) pressure than is PFP operationalized as a proportion, amount, or adoption*.

### National culture as a moderator of the PFP-outcomes relationships

Culture shapes the way people think and behave. Influenced by countries or regions, different national cultures have been formed during the development of human civilization. National cultures distinguish people of the same national culture from others (Hofstede et al., 2010). As a pivotal national culture characteristic, Individualism-collectivism may moderate the relationships between PFP and employee outcomes. People care more about the interest of the group they belong to in collectivistic countries than in individualistic countries in which people consider their interests (Hofstede et al., 2010). For collective interest and harmony, collectivist countries, such as China, Japan, and Korea, emphasize equalization or reduction of differences in pay distribution (Li and Hu, 2012; He and Fang, 2016). However, a growing number of studies with samples of employees in collectivistic countries have found that employees in collectivist countries tend to be positively motivated by PFP, despite this pay system conflicts with their national culture (Chang, 2002; Chang and Hahn, 2006; Du and Choi, 2009; Zhang et al., 2021). These conclusions suggest that equal pay distribution or fewer pay differentials do not adequately motivate employees. In such a situation of input-output inequality, once PFP is added to compensation management, its incentive effects for employees in collectivist countries will be significantly higher than those in individualist countries (Chang, 2002; He and Fang, 2016). Thus, PFP plays a stronger positive role in collectivist countries than individualist countries.

Moreover, national culture may provide a reasonable explanation for the inconsistent findings on the relationship between PFP and extra-role behavior (e.g., helping behaviors, organizational citizenship behavior, etc.). PFP means that pay is determined by performance, so employees devote more effort to in-role issues and reduce the effort to extra-role issues to maximize their pay. However, compared with employees in individualistic countries who place individual interests above the organization, employees in collectivistic countries are more likely to sacrifice their interests to benefit the organization. As a result, employees in collectivistic countries may tend to do more extra-behaviors that are not required but beneficial to the organization, i.e., contextual performance. Combined, we hypothesize that,

*Hypotheses 7a-7g: The positive associations between PFP and (a) task performance, (b) contextual performance, (c) distributive justice, (d) procedural justice, (e) pay satisfaction, (f) intrinsic motivation, and (g) pressure are stronger in samples from collectivistic countries than they are in samples from individualistic countries*.

## Methods

### Literature search

To identify studies that could be used in our meat-analysis, we first searched for articles and dissertations published before November 2021 in ISI Web of Science and ProQuest. The search terms we used are “pay for performance,” “performance pay,” “variable pay,” “performance-related pay,” “performance-contingent pay,” “pay contingent on performance,” “contingent pay,” “performance-based pay,” and “output-based pay.” We also searched by replacing the word “pay” with “compensation,” “wage,” “incentive,” “income,” “bonus” and “reward” in the above terms. Second, we conducted a CNKI (one of the largest Chinese citation databases) search using the same search terms in Chinese. Third, we checked the references of previous reviews about PFP to find articles that were not included during the database searches. Finally, we searched for and included PFP-themed papers from recent Academy of Management conferences to obtain unpublished papers. In addition, we also made an effort to obtain unpublished articles by requesting working papers from colleagues in the field of PFP research.

### Inclusion/exclusion criteria

**Figure 1:**
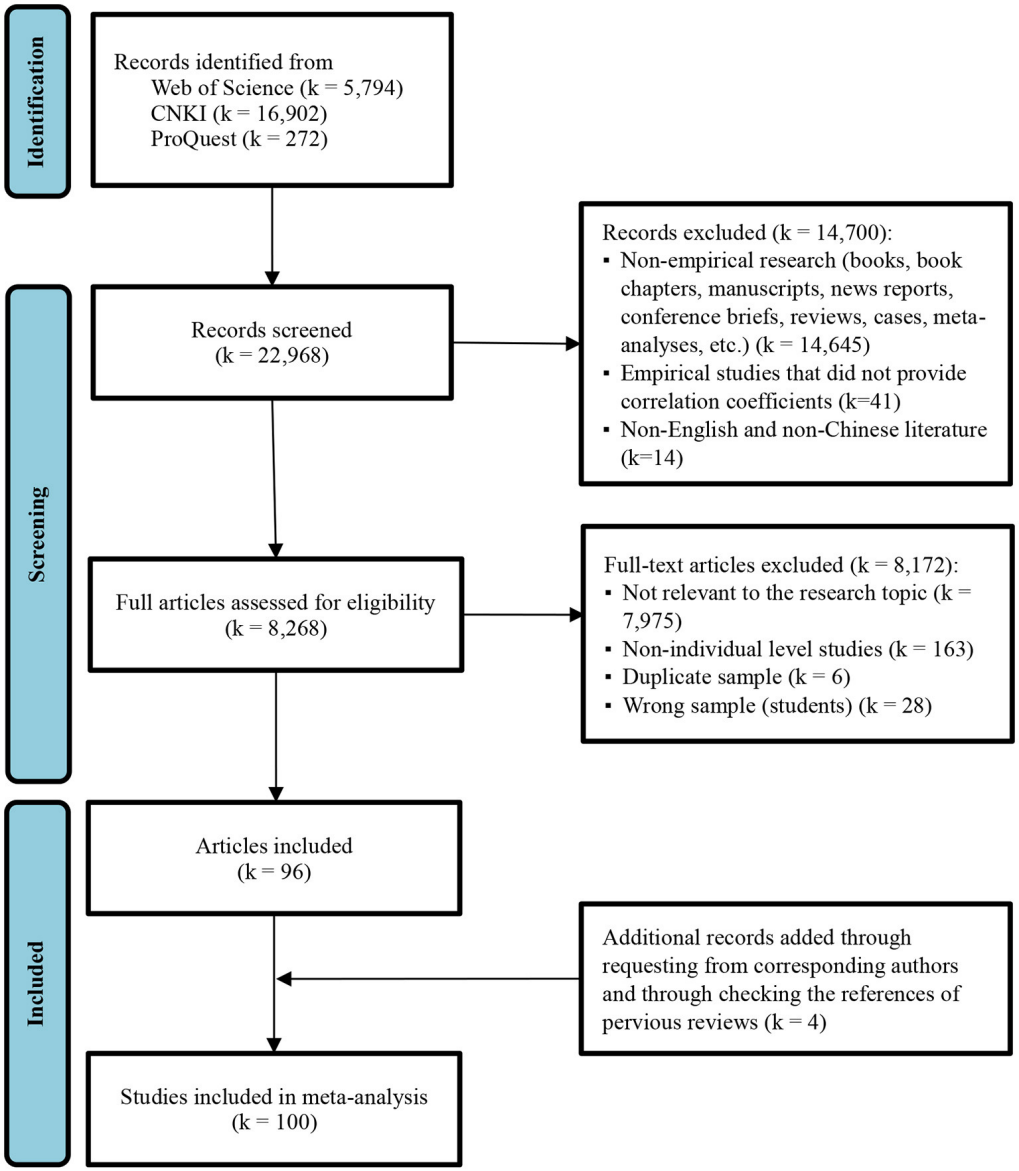
Flowchart of the study selection process.

We used six criteria to evaluate whether a study was included or not in the meta-analysis. First, a study had to be an empirical analysis, and we only included empirical studies that provided at least one correlation between PFP and our other variables of interest. We excluded articles that contain only regression coefficients, as other variables influence the regression coefficients in models, which may distort the correlation relationship between the variables. Second, a study had to report sample size for us to calculate a sample weighted effect size. Third, if a study reports two or more independent samples, we coded these independent samples separately. On the contrary, if the same sample was used in two or more articles, we only considered the article that offered more information. Forth, we focused only on studies that examined PFP at the individual levels. We excluded studies investigating the relationship between CEO PFP and individual behaviors. Fifth, we used only those studies conducted in workplace settings. We excluded those studies with a sample of students (e.g., Rack et al., 2011; Belogolovsky and Bamberger, 2014). Finally, if the articles are published repeatedly, only one of them shall be selected. We only included the published journal article if the dissertation is revised and published in a journal. These inclusion criteria resulted in a final set of 100 articles representing 108 independent samples (*N* = 71,438). The PRISMA flowchart of the study review and selection process is shown in Figure 1.

### Coding procedure

We first created a coding table including coders' names, the basic article information (e.g., authors name, published year, journal name), effect size information (e.g., correlation coefficient, sample size, key variables, reliability of variables, and moderator data (e.g., country, measurement of key variables, etc.). Next, after developing the coding guidelines, the first author and two research assistants independently coded a random selection of 10 articles, and then discussed and settled their disagreements. After two research assistants coded the rest of the articles and discussed any ambiguities with the first author to achieve an agreement, the first author checked all the coding data and resolved errors.

We coded job performance using Borman and Motowidlo (1993) definition, which classified job performance into two categories: task performance and contextual performance. When coding job performance, we pay particular attention to the different ways of expressing performance. For example, task performance includes task performance, in-role performance (behavior), sale performance, work performance, and individual performance, whereas contextual performance includes contextual performance, relational performance, extra-role performance (behavior), organizational citizenship behavior (OCB), helping behavior, and voice behavior. Justice was operationalized as employees' subjective perceptions of the fairness of their input-to-output ratio and was classified into distributive justice and procedural justice based on the typology proposed by Cropanzano et al. (2001). Intrinsic motivation is referred to as motivation that draws people into an activity out of interest and enjoyment in the activity itself. Given its relevance to pay theories, we only coded the pay satisfaction version of job satisfaction. Measures of pressure included pressure, performance pressure, pressure to produce, and stress.

For the moderator, we use scores from Hofstede et al. (2010)'s Cultural Values Survey to code national culture. The survey rates countries or regions according to their individualistic tendencies, with higher scores indicating stronger individualistic tendencies and weaker collectivistic tendencies, and lower scores indicating stronger collectivistic tendencies and weaker individualistic tendencies. Consistent with Rockstuhl et al. (2012), we used the median split of individualism scores to categorize the studies into individualistic and collectivistic countries. In this study, when the sample source region scored below 40 in terms of individualistic tendencies, we labeled it as a collectivistic country, with representative countries or regions such as South Korea, China (including Mainland China, Hong Kong, Macao and Taiwan), Portugal, Turkey, etc. When the score of individualistic tendencies exceeded 40, we labeled it as individualistic countries, with representative countries or regions Germany, Canada, USA, Israeli, etc.

To code the PFP operationalization, we first examined each PFP construct's measure items, looking for each measurement's differences and similarities. We then categorized the PFP measures into four categories: perception, proportion, amount, and adoption. We coded the measure as “perception” if the PFP construct was measured as the association between performance and pay perceived by the employee (Deckop et al., 1999; Ren et al., 2017; Yang et al., 2019). It was coded as “proportion” if it was measured as the proportion of performance-based pay in total pay, regardless of the report from the employees themselves or the calculation based on the organizational archival records (Du and Choi, 2009; Shi et al., 2016). It was coded as “amount” if measured as the actual data on the amount of performance-based pay (Kuvaas et al., 2020a,b). It was coded as “adoption” if it was measured as the adoption of a pay system in which pay changes with performance instead of fixed pay (Shantz et al., 2018; Park and Yang, 2019). Measures that were unclear, varying, or had a mix of two or more measurements mentioned above were coded as “unclear” and were not included in the analysis of the moderating effect of PFP operationalization.

### Meta-analysis procedure

We employed the meta-analytic approach suggested by Hunter and Schmidt (2004), which is widely used in organizational management research. Specially, we adapted random-effects meta-analysis, because the random-effect model reflects the regularity of the organizational management science and tends to be more consistent with reality, compared with the fixed-effect model.

#### Compositing effect sizes

When the study included multiple dimensions of the independent or dependent variables [e.g., Fong and Shaffer (2003) separated PFP into instrumentality perception and expectancy perception, and pay satisfaction into pay level satisfaction, pay structure satisfaction, pay rise satisfaction, and group incentive plan satisfaction], we synthesized the effect sizes of multidimensional variables according to the formula proposed by Hunter and Schmidt (2004).

#### Correcting effect sizes

We used Cronbach's alpha coefficients to correct correlation coefficients obtained in each study for unreliability. If studies didn't report reliabilities, we replaced the missing reliabilities based on two principles. For variables measured by archival data (e.g., task performance ratings from organizational records), we adopted a more conservative 0.80 to replace the missing values of reliabilities, which have been used in many meta-analysis studies (Dalton et al., 2003; Jiang et al., 2012). Also, we recalculated the reliabilities of such variables with 1.00, and the results were the same. For variables measured by subjective data, we replaced the missing values of reliabilities with the sample-weighted average reliabilities estimated from other studies (Lipsey and Wilson, 2001).

#### Calculating effect sizes and other related parameters

We calculated the mean sample-size-weighted observed correlation (r), and the mean sample-size-weighted corrected correlation (ρ). We also computed the number of studies (k), the total sample size (N), the standard deviation (*SD*_ρ_), 95% confidence interval (95% CI), and 80% credibility interval (80% CV) around the mean sample-size-weighted corrected correlation. Moreover, we unitized the Q statistic to evaluate the heterogeneity of a given relationship and the fail-safe number (*N*_*fs*_) to detect publication bias.

#### Tests of moderation and mediation

For the moderator analyses, we estimated the mean sample-size-weighted correlated correlation for each moderator subgroup. As recommended by Chiaburu et al. (2013), we then used Z-scores to determine whether the corrected correlations were significantly different from each subgroup. For the mediating mechanism examinations, we used meta-analytic structural modeling (MASEM) to investigate the potential pathways of PFP on job performance and estimated our proposed mediation models (Viswesvaran and Ones, 1995). First, we created the correlation coefficient matrix. The correlation coefficients between PFP and mediators and outcome variables were calculated based on the data collected in our study. The correlation coefficients between mediators and correlation coefficients between mediators and outcome variables were obtained from previous meta-analysis studies. For correlation coefficients that could not be found in previously published meta-analysis articles, we conducted an additional coding and computed the mean sample-size-weighted corrected correlation following the procedures we listed in this article (e.g., the matrix values of pay satisfaction on contextual performance). Next, we utilized five established model fit statistics to evaluate the path model fit to the data, such as chi-square (χ^2^), the root-mean-square error of approximation (RMSEA), comparative fit index (CFI), tucker-lewis index (TLI), and standardized root-mean-square residual (SRMR). Finally, we reported the path coefficients (β), the indirect effect estimates, and the 95% confidence interval of the indirect effect.

## Results

### Main effects of PFP

Before hypothesis testing, we conducted publication bias tests on the outcome variables related to PFP involved in the study. Since most journals are currently biased to publish articles with significant results, this may prevent articles without significant findings from being included in the meta-analytic study sample, which could easily affect the accuracy of the meta-analytic results (Hunter and Schmidt, 2004). The fail-safe N (*N*_*fs*_) was proposed by Rosenthal (1979) as a useful indicator of publication bias, indicating the minimum number of unpublished studies needed to reverse the findings of a meta-analysis to exclude the possibility of publication bias. When the *N*_*fs*_ is >5k+10 (k denotes the number of independent samples), the larger the value, the more stable the analysis results are and the less likely there is a publication bias problem. When the *N*_*fs*_ is < 5k+10, it indicates that publication bias needs to be taken seriously (Rosenthal, 1979; Rhoades and Eisenberger, 2002). Table 1 presented the *N*_*fs*_ for the relationships between PFP and other variables at a *p*-value of 0.05. The *N*_*fs*_ for all relationships were much > 5k+10, indicating that the overall findings of this study were stable and there was little possibility of publication bias.

**Table 1:** Meta-analytic results for PFP, job performance and other individual outcomes.

**Variable**	**k**	***N***	***r***	***SD*_*r*_**	**ρ**	***SD*_*p*_**	***CI*_*LL*_**	***CI*_*UL*_**	***CV*_*LL*_**	***CV*_*UL*_**	**Q**	***N*_*fs*_**
Job performance	48	40,939	0.20	0.16	0.23	0.19	0.18	0.29	−0.13	0.48	1189.60^**^	14,719
Task performance	29	34,833	0.22	0.14	0.26	0.17	0.19	0.32	0.04	0.47	771.41^**^	9,282
Contextual performance	28	7,740	0.14	0.25	0.17	0.28	0.06	0.28	−0.19	0.53	476.39^**^	2,206
Intrinsic motivation	20	5,936	0.11	0.14	0.14	0.15	0.07	0.21	−0.06	0.33	115.54^**^	701
Pressure	5	2,421	0.13	0.12	0.18	0.15	0.04	0.32	−0.01	0.37	35.12^**^	112
Distributive justice	16	4,577	0.31	0.24	0.36	0.28	0.22	0.50	0.00	0.72	324.25^**^	3,325
Procedural justice	24	5,624	0.28	0.24	0.34	0.28	0.22	0.45	−0.02	0.70	375.27^**^	4,931
Pay satisfaction	26	19,591	0.14	0.19	0.16	0.22	0.08	0.25	−0.12	0.45	700.55^**^	8,960

k = number of studies; N = total sample size; r = sample-size weighted mean uncorrected correlation; *SD*_*r*_ = observed standard deviation of uncorrelated correlations; ρ = sample-size weighted mean corrected correlation, corrected for unreliability; *SD*_ρ_ = corrected standard deviation of corrected correlations; *CI*_*LL*_ and *CI*_*UL*_: lower and upper bounds, respectively, of the 95% confidence intervals around the corrected mean correlations; *CV*_*LL*_ and *CV*_*UL*_: lower and upper bounds, respectively, of the 80% credibility intervals; Q = corrected correlation heterogeneity; *N*_*fs*_ = number of unlocated studies with non-significant effect sizes required to render the ρ non-significant.

^*^p < 0.05, ^**^p < 0.01.

The meta-analytic results for the relationships between PFP and other variables can be found in Table 1. As shown in Table 1, PFP had a positive effect on job performance (ρ = 0.23, 95% CI = [0.18, 0.29]), and PFP had a stronger association with task performance (ρ = 0.26, 95% CI = [0.19, 0.32]) than with contextual performance (ρ = 0.17, 95% CI = [0.06, 0.28]). Therefore, Hypothesis 1a and 1b were both supported.

In addition to the meta-analytic correlations between PFP and job performance, Table 1 also presented the meta-analytic results of PFP with other individual outcomes. Results showed that PFP had positive effects on intrinsic motivation (ρ = 0.14, 95% CI = [0.07, 0.21]), pressure (ρ = 0.18, 95% CI = [0.04, 0.32]), distributive justice (ρ = 0.36, 95% CI = [0.22, 0.50]), procedural justice (ρ = 0.34, 95% CI = [0.22, 0.45]), and pay satisfaction (ρ = 0.16, 95% CI = [0.08, 0.25]). In sum, Hypotheses 2a-2b and Hypotheses 4a-4c were supported.

### Mediating effects of intrinsic motivation and pressure

Hypothesis 3 predicted that the relationship between PFP and job performance (e.g., task performance and contextual performance) would be mediated by intrinsic motivation and pressure. To test these hypotheses, we put the correlation matrix (Table 2) into Mplus 8.0 by conducting a MASEM. As Viswesvaran and Ones (1995) suggested, we used the harmonic mean as the sample size for the structural equation model (in this model, *N* = 6,054). The resulting model fit the data adequately well: χ^2^ (1, *N* = 6,054) = 0.17, CFI = 1.00; TLI = 1.00, RMSEA = 0.00, SRMR = 0.002.

**Table 2:** Correlations among cognitive evaluation and job performance outcomes.

	**1**	**2**	**3**	**4**
1. PFP	**–**			
2. task performance ($\bar{r}$	0.22, 0.26			
k, N	29, 34833			
3. contextual performance ($\bar{r}$	0.14, 0.17	0.20, 0.23^a^		
k, N	28, 7740	24, 9912		
4. intrinsic motivation ($\bar{r}$	0.11, 0.14	0.30, 0.36^b^	0.26, 0.31^b^	
k, N	20, 5936	43, 21200	16, 12259	
5. pressure ($\bar{r}$	0.13, 0.18	−0.03, −0.05^c^	−, −0.01^d^	0.01, 0.02^c^
k, N	5, 2421	47, 9204	14, 5065	9, 2654

k = number of studies; N = total sample size; r = sample-size weighted mean uncorrected correlation; ρ = sample-size weighted mean corrected correlation, corrected for unreliability.

^a^ρ form Harrison et al. (2006); ^b^ρ form Van den Broeck et al. (2021); ^c^ρ form LePine et al. (2005); ^d^ρ from Zhang et al. (2019) (sample-size weighted mean uncorrected correlation not provided). All meta-analytic estimates that appear without a superscript are original analyses.

**Figure 2:**
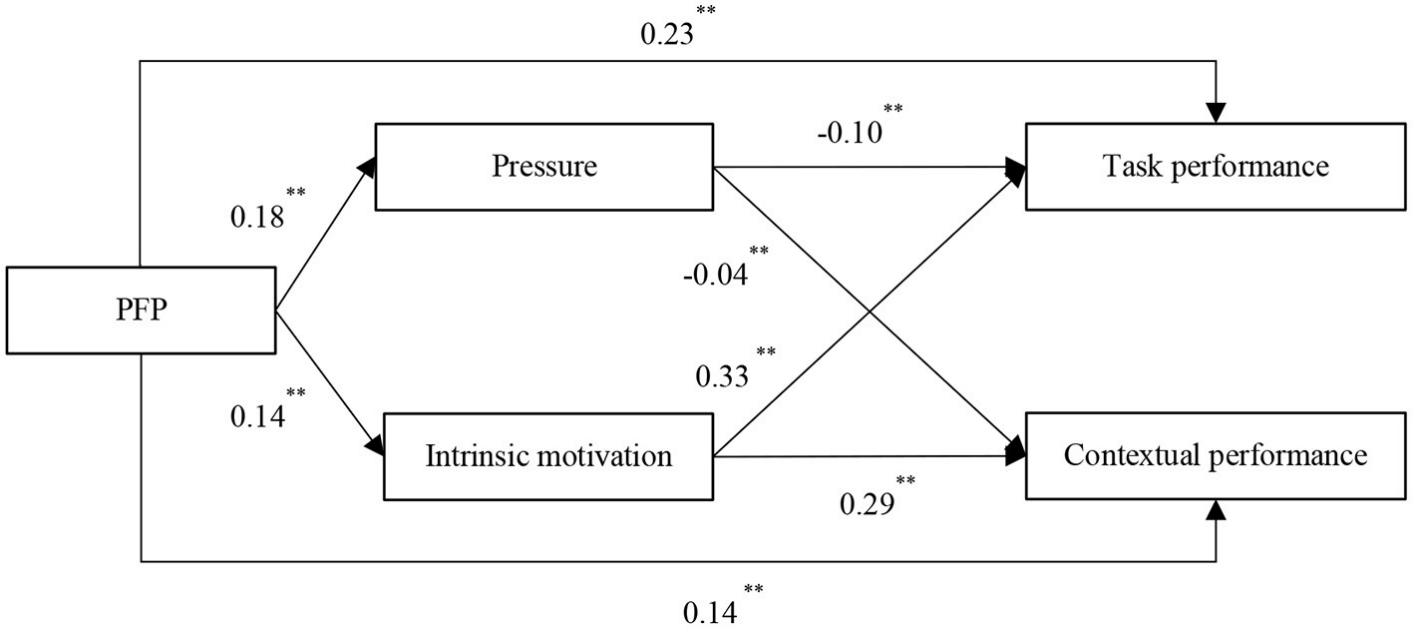
Structural equation modeling results with cognitive evaluation. Coefficients presented were unstandardized estimates. Harmonic *N* = 6,054, *p < 0.05, **p < 0.01.

Figure 2 illustrated that PFP was positively related to pressure (β = 0.18, *p* < 0.01) and intrinsic motivation (β = 0.14, *p* < 0.01). The pressure was a significant negative predictor of task performance (β = −0.10, *p* < 0.01), and intrinsic motivation was a significant positive predicator of it (β = 0.33, *p* < 0.01). For contextual performance, pressure (β = −0.04, *p* < 0.01) and intrinsic motivation (β = 0.29, *p* < 0.01) had opposite effects on contextual performance.

As shown in Table 3, PFP had a significant indirect relationship with task performance, mediated through both pressure (indirect effect = −0.02, 95% CI = [−0.02, −0.01]) and intrinsic motivation (indirect effect = 0.05, 95% CI = [0.04, 0.06]). Similarly, the relationship between PFP and contextual performance was mediated by both pressure (indirect effect = −0.01, 95% CI = [−0.01, −0.00]) and intrinsic motivation (indirect effect = 0.04, 95% CI = [0.03, 0.05]). These results offer support for Hypothesis 3.

**Table 3:** Tests of mediation for cognitive evaluation.

**Path**	**Effect**	**SE**	**95% CI**
**Indirect effect**			
IND1: PFP → PR → TP	−0.02^**^	0.00	[−0.02, −0.01]
IND2: PFP → PR → CP	−0.01^**^	0.00	[−0.01, −0.00]
IND3: PFP → IM → TP	0.05^**^	0.00	[0.04, 0.06]
IND4: PFP → IM → CP	0.04^**^	0.00	[0.03, 0.05]
**Indirect effect difference**			
IND1-IND3: PFP → PR → TP- PFP → IM → TP	−0.06^**^	0.01	[−0.07, −0.05]
IND2-IND4: PFP → PR → CP- PFP → IM → CP	−0.05^**^	0.00	[−0.06, −0.04]
**Direct effect**			
PFP → TP	0.23^**^	0.01	[0.21, 0.26]
PFP → CP	0.14^**^	0.01	[0.11, 0.16]
**Total effect**			
PFP → TP	0.26^**^	0.01	[0.24, 0.29]
PFP → CP	0.17^**^	0.01	[0.14, 0.20]

PFP, pay for performance; PR, pressure; IM, intrinsic motivation; TP, task performance; CP, contextual performance.

Estimates were tested for significance using bias-corrected confidence intervals from 20,000 resamples through the R program.

N = 6,054. ^*^p < 0.05, ^**^p < 0.01.

### Mediating effects of distributive justice and procedural justice

In Hypothesis 5, we proposed that justice (e.g., distributive justice, procedural justice) will mediate the influence of PFP on task performance and contextual performance. To test this prediction, we followed the testing procedure as with Hypothesis 3. We input the correlation matrix (Table 4) into Mplus 8.0 by conducting MASEM, and the model resulted in an acceptable fit to the data (X^2^ (1, *N* = 4,401) = 21.13, CFI = 0.998; TLI = 0.967, RMSEA = 0.068, SRMR = 0.010).

**Table 4:** Correlations among justice and job performance outcomes.

**Variable**	**1**	**2**	**3**	**4**	**5**
1. PFP	**—**				
2. task performance (*r*, ρ)	0.22, 0.26				
k, N	29, 34833				
3. contextual performance (*r*, ρ)	0.14, 0.17	0.20, 0.23^a^			
k, N	28, 7740	24, 9912			
4. pay satisfaction (*r*, ρ)	0.14, 0.16	0.03, 0.05^b^	0.14, 0.18		
k, N	26, 19591	43, 14848	3, 583		
5. distributive justice (*r*, ρ)	0.31, 0.36	0.19, 0.26^c^	0.17, 0.21^c^	0.61, 0.79^b^	
k, N	16, 4577	45, 11336	36, 10100	10, 6595	
6.procedural justice (*r*, ρ)	0.28, 0.34	0.19, 0.24^c^	0.23, 0.30^c^	0.36, 0.42^b^	0.51, 0.61^c^
k, N	24, 5624	57, 14258	71, 16864	8, 2291	184, 67956

k = number of studies; N = total sample size; r = sample-size weighted mean uncorrected correlation; ρ = sample-size weighted mean corrected correlation, corrected for unreliability.

^a^ρ form Harrison et al. (2006); ^b^ρ form Williams et al. (2006); ^c^ρ form Colquitt et al. (2013).

**Figure 3:**
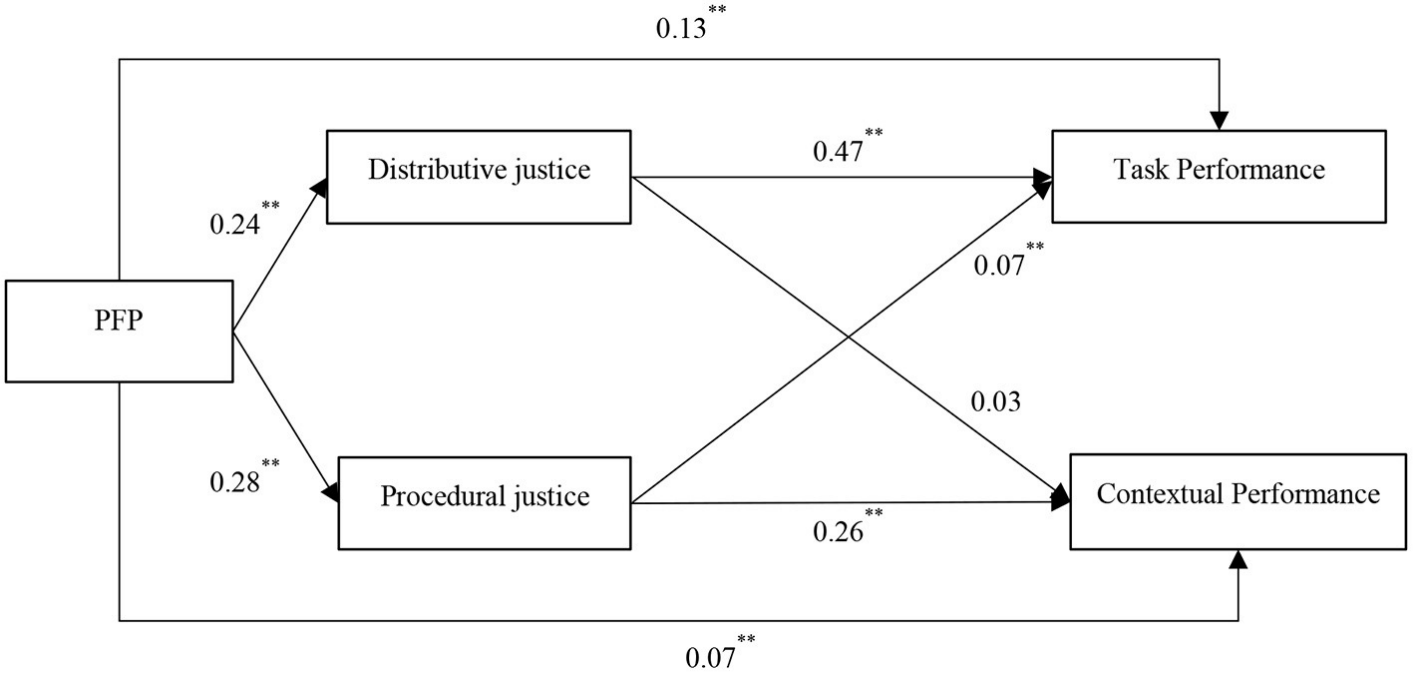
Structural equation modeling results with justice. Coefficients presented were unstandardized estimates. Harmonic *N* = 4,401, *p < 0.05, **p < 0.01.

Figure 3 presents the results of the path analysis of the influence of PFP on job performance (*via* justice). We controlled for pay satisfaction (not shown in Figure 3) because previous research has found pay satisfaction to influence employees' justice perceptions and task performance (Colquitt et al., 2013). As shown in Figure 3, PFP had significant positive effects on distributive justice (β = 0.24, *p* < 0.01) and procedural justice (β = 0.28, *p* < 0.01). Distributive justice had a significant relationship with task performance (β = 0.47, *p* < 0.01), but not contextual performance (β = 0.03, *ns*). Procedural justice had significant unique effects on job performance: procedural justice had a larger and positive relationship with contextual performance (β = 0.26, *p* < 0.01), as compared to task performance (β = 0.07, *p* < 0.01).

Table 5 reports the indirect and total effects of PFP on individual job performance through distributive justice and procedural justice. As shown in Table 5, PFP had a significant indirect relationship with task performance, mediated through both distributive justice (indirect effect = 0.11, 95% CI = [0.10, 0.13]) and procedural justice (indirect effect = 0.02, 95% CI = [0.01, 0.03]). Compared to these two mediating results, the relationship between PFP and task performance may be more explained by distributive justice than procedural justice. Table 5 also indicated that procedural justice positively mediated the relationship between PFP and contextual performance (indirect effect = 0.07, 95% CI = [0.06, 0.09]). In contrast, distributive justice did not play a significant mediating role between PFP and contextual performance (indirect effect = 0.01, 95% CI = [**–**0.00, 0.01]). In other words, the primary mediating by which PFP affects contextual performance was procedural justice. Thus, Hypothesis 5 is partially supported.

**Table 5:** Tests of mediation for justice.

**Path**	**Effect**	**SE**	**95% CI**
**Indirect effect**			
IND1: PFP → DJ → TP	0.11^**^	0.01	[0.10, 0.13]
IND2: PFP → DJ → CP	0.01	0.00	[−0.00, 0.01]
IND3: PFP → PJ → TP	0.02^**^	0.01	[0.01, 0.03]
IND4: PFP → PJ → CP	0.07^**^	0.01	[0.06, 0.09]
**Indirect effect difference**			
IND1-IND3: PFP → DJ → TP- PFP → PJ → TP	0.09^**^	0.01	[0.08, 0.11]
IND2-IND4: PFP → DJ → CP- PFP → PJ → CP	−0.07^**^	0.01	[−0.08, −0.05]
**Direct effect**			
PFP → TP	0.13^**^	0.01	[0.10, 0.16]
PFP → CP	0.07^**^	0.02	[0.04, 0.10]
**Total effect**			
PFP → TP	0.26^**^	0.02	[0.22, 0.29]
PFP → CP	0.15^**^	0.02	[0.12, 0.19]

PFP, pay for performance; DJ, distributive justice; PJ, procedural justice; TP, task performance; CP, contextual performance.

Estimates were tested for significance using bias-corrected confidence intervals from 20,000 resamples through the R program.

N = 4,401. ^*^p < 0.05, ^**^p < 0.01.

### Moderating effects of PFP operationalization

Hypotheses 6a-6g predicated that PFP operationalization would be a significant moderator of the PFP-outcomes relationships. Table 6 provided the results for the moderating effect of the PFP measure. As shown in Table 6, studies that operationalized PFP as a perception (ρ = 0.16, 95% CI = [0.10, 0.22]) had significantly smaller effect sizes than studies that operationalized PFP as a proportion (ρ = 0.39, 95% CI = [0.31, 0.46]; Z = 5.02, *p* < 0.01), which evidenced the moderating effect of PFP operationalization on the PFP-task performance relationship, but in the opposite direction to what we hypothesized. Thus, Hypothesis 6a was partially supported.

**Table 6:** Moderating effect of PFP operationalization on relationships between PFP and outcomes.

**Variable**	**k**	***N***	***r***	***SD*_*r*_**	**ρ**	***SD*_*p*_**	***CI*_*LL*_**	***CI*_*UL*_**	***CV*_*LL*_**	***CV*_*UL*_**	**Q**	**Z**
**Task performance**												
Proportion	8	14,900	0.31	0.08	0.39	0.10	0.31	0.46	0.26	0.51	126.61^**^	Z = 5.02, *p < * 0.01
Perception	17	17,604	0.13	0.11	0.16	0.12	0.10	0.22	0.00	0.31	203.46^**^	
**Contextual performance**												
Proportion	10	3,049	−0.01	0.17	−0.01	0.19	−0.13	0.12	−0.25	0.24	86.74^**^	Z = −2.94, *p < * 0.01
Perception	17	4,572	0.23	0.24	0.28	0.28	0.15	0.42	−0.07	0.64	296.52^**^	
**Distributive justice**												
Proportion	4	1,369	0.10	0.13	0.12	0.15	−0.04	0.29	−0.08	0.32	26.42^**^	Z = −3.10, *p < * 0.01
Perception	12	3,208	0.39	0.22	0.46	0.26	0.30	0.61	0.12	0.79	232.52^**^	
**Procedural justice**												
Proportion	6	1,583	0.13	0.08	0.16	0.06	0.08	0.24	0.08	0.24	9.89^†^	Z = −3.57, *p < * 0.01
Perception	16	3,465	0.38	0.24	0.46	0.29	0.31	0.60	0.08	0.83	289.42^**^	
**Pay satisfaction**												
Proportion	5	1,942	0.11	0.13	0.13	0.14	0.00	0.26	−0.05	0.31	32.36^**^	Z_pr − pe_ = −3.99, *p < * 0.01
Perception	16	5,263	0.38	0.16	0.45	0.18	0.36	0.55	0.22	0.69	182.64^**^	Z_pr − ad_ = 1.66, *p =* 0.10
Adoption	2	11,712	0.02	0.00	0.02	0.00	0.01	0.04	0.02	0.02	1.28	Z_pe − ad_ = 9.06, *p < * 0.01
**Intrinsic motivation**												
Proportion	3	683	0.08	0.06	0.10	0.00	0.02	0.17	0.10	0.10	2.10	Z_pr − pe_= −1.96, *p =* 0.05
Perception	13	3,679	0.17	0.13	0.21	0.14	0.13	0.30	0.04	0.39	60.81^**^	Z_pr − am_ = 1.54, *p =* 0.12
Amount	3	1,114	−0.02	0.11	−0.03	0.11	−0.17	0.11	−0.17	0.11	12.87^**^	Z_pr − ad_ = 1.63, *p =* 0.10
Adoption	1	460	0.03	0.00	0.04	0.00	0.04	0.04	0.04	0.04	–	Z_pe − am_ = 2.81, *p < * 0.01
												Z_pe − ad_ = 3.97, *p < * 0.01
												Z_am − ad_ = −0.89, *p =* 0.37
**Pressure**												
Proportion	2	520	0.26	0.13	0.30	0.14	0.09	0.51	0.13	0.48	10.01^**^	Z = 1.66, *p* = 0.10
Perception	2	1,619	0.06	0.08	0.09	0.10	−0.07	0.24	−0.04	0.24	9.61^**^	

k = number of studies; N = total sample size; r = sample-size weighted mean uncorrected correlation; *SD*_*r*_ = observed standard deviation of uncorrelated correlations; ρ = sample-size weighted mean corrected correlation, corrected for unreliability; *SD*_ρ_ = corrected standard deviation of corrected correlations; *CI*_*LL*_ and *CI*_*UL*_: lower and upper bounds, respectively, of the 95% confidence intervals around the corrected mean correlations; *CV*_*LL*_ and *CV*_*UL*_: lower and upper bounds, respectively, of the 80% credibility intervals; Q = corrected correlation heterogeneity; in Z-test, subscripts pr, pe, am, and ad is abbreviations for proportion, perception, amount, and adoption, respectively.

^†^*p* < 0.1, ^*^p < 0.05, ^**^p < 0.01.

As expected (Hypothesis 6b-6d), PFP operationalized as a perception was more strongly related to contextual performance (perception: ρ = 0.28, 95% CI = [0.15, 0.42]; proportion: ρ = **–**0.01, 95% CI = [−0.13, 0.12], Z = −2.94, *p* < 0.01), distributive justice (perception: ρ = 0.46, 95% CI = [0.30, 0.61]; proportion: ρ = 0.12, 95% CI = [−0.04, 0.29], Z = −3.10, *p* < 0.01), and procedural justice (perception: ρ = 0.46, 95% CI = [0.31, 0.60]; proportion: ρ = 0.16, 95% CI = [0.08, 0.24], Z = −3.57, *p* < 0.01) than PFP operationalized as a proportion.

With Hypothesis 6e, our results indicated that PFP operationalized as a perception is most strongly associated with pay satisfaction (ρ = 0.45, 95% CI = [0.36, 0.55]) compared with PFP operationalized as a proportion (ρ = 0.13, 95% CI = [0.00, 0.26]; Z = −3.99, *p* < 0.01) and adoption (ρ = 0.02, 95% CI = [0.01, 0.04]; Z = 9.06, *p* < 0.01).

For Hypothesis 6f, Table 6 illustrated that PFP operationalized as a perception to be most strongly associated with intrinsic motivation (ρ = 0.21, 95% CI = [0.13, 0.30]) compared with PFP operationalized as an amount (ρ = −0.03, 95% CI = [−0.17, 0.11]; Z = 2.81, *p* < 0.01) and adoption (ρ = 0.04, 95% CI = [0.04, 0.04]; Z = 3.97, *p* < 0.01). However, there was no significant difference in effect sizes between studies that operationalized PFP as a perception and studies that operationalized PFP as a proportion (ρ = 0.10, 95% CI = [0.02, 0.17]; Z = −1.96, *p* = 0.05). Thus, it offered partial support for Hypothesis 6f. For Hypothesis 6g, Table 6 showed that the PFP operationalization did not moderate the relationship between PFP and pressure, as there was no significant difference between studies using perception (ρ = 0.09, 95% CI = [−0.07, 0.24]) and studies using proportion (ρ = 0.30, 95% CI = [0.09, 0.51]; Z = 1.66, *p* = 0.10). Thus, hypothesis 6g was not supported.

### Moderating effects of national culture

Hypotheses 7a-7g predicated that national culture would be a significant moderator of the PFP-outcomes relationships. Table 7 provided the results for the moderating effect of national culture. Our results showed that national culture failed to moderate the PFP-task performance relationship, as there was no significant different in effect sizes between studies from individualistic countries and studies from collectivistic countries (individualism: ρ = 0.25, 95% CI = [0.16, 0.33]; collectivism: ρ = 0.32, 95% CI = [0.22, 0.42], Z = −1.05, *p* = 0.29). Therefore, Hypothesis 7a was not supported.

**Table 7:** Moderating effect of national culture on relationships between PFP and outcomes.

**Variable**	**k**	***N***	***r***	***SD*_*r*_**	**ρ**	***SD*_*p*_**	***CI*_*LL*_**	***CI*_*UL*_**	***CV*_*LL*_**	***CV*_*UL*_**	**Q**	**Z**
**Task performance**												
Individualism	14	30,840	0.21	0.13	0.25	0.16	0.16	0.33	0.04	0.46	627.63^**^	Z = −1.05, *p =* 0.29
Collectivism	15	3,993	0.27	0.17	0.32	0.19	0.22	0.42	0.07	0.56	134.11^**^	
**Contextual performance**												
Individualism	9	2,814	0.02	0.16	0.03	0.17	−0.09	0.15	−0.19	0.25	70.14^**^	Z = −2.59, *p < * 0.01
Collectivism	17	4,011	0.24	0.28	0.31	0.32	0.15	0.46	−0.10	0.71	323.75^**^	
**Distributive justice**												
Individualism	1	367	−0.10	0.00	−0.11	0.00	−0.11	−0.11	−0.11	−0.11	–	Z = −8.00, *p < * 0.01
Collectivism	13	3,544	0.38	0.21	0.44	0.25	0.30	0.58	0.12	0.77	234.76^**^	
**Procedural justice**												
Individualism	5	1,198	0.12	0.10	0.15	0.09	0.05	0.25	0.04	0.26	11.75^*^	Z = −3.08, *p < * 0.01
Collectivism	17	3,760	0.35	0.25	0.43	0.30	0.28	0.57	0.05	0.81	311.40^**^	
**Pay satisfaction**												
Individualism	5	12,316	0.04	0.08	0.05	0.10	−0.04	0.13	−0.08	0.17	84.02^**^	Z = −4.60, *p < * 0.01
Collectivism	20	7,183	0.30	0.19	0.36	0.22	0.26	0.46	0.07	0.65	321.78^**^	
**Intrinsic motivation**												
Individualism	5	1,607	0.04	0.13	0.05	0.15	−0.09	0.19	−0.14	0.24	29.39^**^	Z = −1.42, *p =* 0.15
Collectivism	14	3,923	0.13	0.14	0.17	0.15	0.08	0.25	−0.02	0.36	72.41^**^	
**Pressure**												
Individualism	3	527	0.22	0.11	0.26	0.14	0.07	0.45	0.08	0.44	9.93^*^	Z = 0.75, *p =* 0.45
Collectivism	2	1,894	0.10	0.12	0.15	0.14	−0.05	0.36	−0.03	0.33	22.61^**^	

k = number of studies; N = total sample size; r = sample-size weighted mean uncorrected correlation; *SD*_*r*_ = observed standard deviation of uncorrelated correlations; ρ = sample-size weighted mean corrected correlation, corrected for unreliability; *SD*_ρ_ = corrected standard deviation of corrected correlations; *CI*_*LL*_ and *CI*_*UL*_: lower and upper bounds, respectively, of the 95% confidence intervals around the corrected mean correlations; *CV*_*LL*_ and *CV*_*UL*_: lower and upper bounds, respectively, of the 80% credibility intervals; Q = corrected correlation heterogeneity.

^†^*p* < 0.1, ^*^p < 0.05, ^**^p < 0.01.

Consistent with our hypotheses 7b-7e, we found that PFP to be more positively related to contextual performance (individualism: ρ = 0.03, 95% CI = [−0.09, 0.15]; collectivism: ρ = 0.31, 95% CI = [0.15, 0.46], Z = −2.59, *p* < 0.01), distributive justice (individualism: ρ = −0.11, 95% CI = [−0.11, −0.11]; collectivism: ρ = 0.44, 95% CI = [0.30, 0.58], Z = −8.00, *p* < 0.01), procedural justice (individualism: ρ = 0.15, 95% CI = [0.05, 0.25]; collectivism: ρ = 0.43, 95% CI = [0.28, 0.57], Z = −3.08, *p* < 0.01), and pay satisfaction (individualism: ρ = 0.05, 95% CI = [−0.04, 0.13]; collectivism: ρ = 0.36, 95% CI = [0.26, 0.46], Z = −4.60, *p* < 0.01) in collectivistic countries than in individualistic countries.

For Hypothesis 7f, although the corrected correlation between PFP and intrinsic motivation in collectivistic countries (ρ = 0.17, 95% CI = [0.08, 0.25]) was greater than the corrected correlation in individualistic countries (ρ = 0.05, 95% CI = [−0.09, 0.19]), the statistic Z is not significant (Z = −1.42, *p* = 0.15). Thus, Hypothesis 7f was not strongly supported. Finally, Hypothesis 7g was not supported, as there was no significant difference in effect sizes between studies from collectivistic countries and studies from individualistic countries (individualism: ρ = 0.26, 95% CI = [0.07, 0.45]; collectivism: ρ = 0.15, 95% CI = [−0.05, 0.36], Z = 0.75, *p* = 0.45).

### Supplemental analysis of publication status

We conducted a *post hoc* analysis to examine whether publication status could be a moderator to explain the variability between studies. As shown in Table 8, the corrected correlation between PFP and task performance in published studies (ρ = 0.35, 95% CI = [0.28, 0.42]) was significantly greater than the corrected correlation in unpublished studies (ρ = 0.13, 95% CI = [0.07, 0.19]; Z = 4.96, *p* < 0.01). There appears to be an upward bias in the published literature on the strength of the relationship between PFP and task performance. However, there was no significant difference in effect sizes between published studies (ρ = 0.11, 95% CI = [−0.01, 0.23]) and unpublished studies in the relationship of PFP and contextual performance (ρ = 0.28, 95% CI = [0.10, 0.47]; Z = −1.48, *p* = 0.14). In the relationship of PFP and distributive justice, our results illustrated that unpublished studies (ρ = 0.57, 95% CI = [0.45, 0.70]) had significantly larger effect sizes than published studies (ρ = 0.19, 95% CI = [0.01, 0.36]; Z = −3.67, *p* < 0.01). Similar results were found in the tests of the relationship between PFP and pay satisfaction (Z = −4.00, *p* < 0.01), and PFP and pressure (Z = −1.97, *p* < 0.05). Thus, publication status was a striking moderator in explaining the variability between these relationships. Finally, our *post hoc* analysis also indicated that there were non-significant differences between published and unpublished studies in the relationship between PFP and procedural justice (Z = −1.24, *p* = 0.21) and the relationship between PFP and intrinsic motivation (Z = −0.58, *p* = 0.56).

**Table 8:** Moderating effect of publication status on relationships between PFP and outcomes.

**Variable**	**k**	***N***	***r***	***SD*_*r*_**	**ρ**	***SD*_*p*_**	***CI*_*LL*_**	***CI*_*UL*_**	***CV*_*LL*_**	***CV*_*UL*_**	**Q**	**Z**
**Task performance**												
Published	22	20,959	0.29	0.13	0.35	0.16	0.28	0.42	0.15	0.55	419.37^**^	Z = 4.96, *p < * 0.01
Unpublished	7	13,874	0.11	0.07	0.13	0.07	0.07	0.19	0.03	0.22	64.43^**^	
**Contextual performance**												
Published	15	4,970	0.09	0.20	0.11	0.23	−0.01	0.23	−0.18	0.40	202.50^**^	Z = −1.48, *p =* 0.14
Unpublished	13	2,770	0.23	0.29	0.28	0.33	0.10	0.47	−0.14	0.71	247.63^**^	
**Distributive justice**												
Published	7	2,515	0.16	0.20	0.19	0.23	0.01	0.36	−0.10	0.48	103.81^**^	Z = −3.67, *p < * 0.01
Unpublished	9	2,062	0.48	0.15	0.57	0.18	0.45	0.70	0.35	0.80	84.27^**^	
**Procedural justice**												
Published	12	2,810	0.22	0.19	0.26	0.21	0.14	0.39	0.00	0.53	102.87^**^	Z = −1.24, *p =* 0.21
Unpublished	12	2,814	0.34	0.27	0.41	0.32	0.22	0.59	−0.01	0.82	264.40^**^	
**Pay satisfaction**												
Published	17	16,909	0.09	0.14	0.11	0.16	0.03	0.19	−0.10	0.32	335.78^**^	Z = −4.00, *p < * 0.01
Unpublished	9	2,682	0.41	0.21	0.48	0.25	0.32	0.65	0.17	0.80	175.36^**^	
**Intrinsic motivation**												
Published	16	4,719	0.10	0.14	0.13	0.15	0.05	0.21	−0.07	0.32	90.65^**^	Z = −0.58, *p =* 0.56
Unpublished	4	1,217	0.15	0.14	0.18	0.15	0.02	0.34	−0.01	0.37	22.98^**^	
**Pressure**												
Published	3	2,079	0.10	0.11	0.15	0.13	−0.01	0.30	−0.02	0.30	22.87^**^	Z = −1.97, *p < * 0.05
Unpublished	2	342	0.29	0.07	0.37	0.09	0.20	0.55	0.26	0.48	3.69^†^	

k = number of studies; N = total sample size; r = sample-size weighted mean uncorrected correlation; *SD*_*r*_ = observed standard deviation of uncorrelated correlations; ρ = sample-size weighted mean corrected correlation, corrected for unreliability; *SD*_ρ_ = corrected standard deviation of corrected correlations; *CI*_*LL*_ and *CI*_*UL*_: lower and upper bounds, respectively, of the 95% confidence intervals around the corrected mean correlations; *CV*_*LL*_ and *CV*_*UL*_: lower and upper bounds, respectively, of the 80% credibility intervals; Q = corrected correlation heterogeneity.

^†^*p* < 0.1, ^*^p < 0.05, ^**^p < 0.01.

## Discussion

### Theoretical implications

First, we deepen the current research's understanding of the relationship between PFP and performance by focusing on those studies that examine it in real work settings. After our literature review, we found that previous meta-analyses of the PFP-performance relationship have included only experimental studies in the analysis (Jenkins et al., 1998; Weibel et al., 2010). The experimental research approach bears a strong trace of artificiality, and the strict control of conditions to a certain extent makes the research context detached from social life reality, which affects the generalization and application of research findings (Rynes and Bono, 2000; Garbers and Konradt, 2014). In fact, unlike experimental studies that mostly consider only the presence or absence of incentives, the actual organizational practice also involves more complex factors such as the design of incentive intensity, pay gap design, and employee cognitive psychology, which can be better reflected in field survey studies. In addition, experimental studies may have greater effects than field survey studies (Henderson and Horan, 2021). By including field survey studies in our meta-analysis, we were able to have a closer approximation to the true PFP-performance relationship and also to correct the mean effect sizes that were inflated by previous studies.

Second, our results confirmed the positive effects of PFP on job performance in the workplace, especially the differential effects on two aspects of performance: task performance and contextual performance. There have been conflicting findings in studies on the effect of PFP on performance, probably because researchers have conflated different aspects of performance. Some studies discussed the impact of PFP on overall performance in an integrated manner, while some articles only explored the role of PFP on task-related performance. They all claimed that they studied the relationship between PFP and performance. Our study not only found a positive effect of PFP on overall job performance but also further distinguished the role of PFP on task performance and contextual performance. Our results indicated a significant positive effect of PFP on both aspects of job performance (i.e., task performance and contextual performance), with the relationship between PFP and task performance being stronger than the relationship between PFP and contextual performance. This finding not only clarifies the differential effect of PFP on in-role performance (task performance) and extra-role performance (contextual performance), but also helps to respond to the current theoretical conflict over the relationship between PFP and job performance. These conclusions shift the debate from “whether PFP motivates employee job performance” to “why PFP affects some performance (task performance) more than others (contextual performance),” which is the issue we are trying to address through MASEM.

Third, based on the cognitive evaluation perspective, we introduced two mediating variables, intrinsic motivation, and pressure, to integrate the positive and negative effects of PFP on job performance in one framework. Previous scholars of PFP research have often one-sidedly emphasized the informational or controlling nature of PFP, ignoring the dual nature of PFP. Studies with a positive view of PFP argue that PFP gives individuals positive feedback that makes them more willing to work hard to achieve their goals. The informational nature of PFP is seen as a facilitator of intrinsic motivation, enhancing individuals' intrinsic interest in work and reducing vigilance against low performance (Deci and Ryan, 1985). Studies with a negative view of PFP argue that PFP undermines individuals' perceptions of the value and meaning of their work, creating a negative effect on intrinsic motivation, or what motivational crowding theory calls a “crowding-out effect” (Frey and Oberholzer-Gee, 1997). This negative effect makes the individual over-focused on the value of the reward and thus stressed performance goals (Fang and Gerhart, 2012). When stressed, employees will perceive themselves as being controlled by the PFP (Deci and Ryan, 1985). The controlling nature of PFP is seen as a catalyst for individual pressure, reducing employees' positive feedback on their work and individual job performance. Our integrative structural equation modeling meta-analysis revealed that the seemingly paradoxical information nature of PFP and the controlling nature of PFP go hand in hand, as PFP can both promote task performance and contextual performance through intrinsic motivation, and reduce task performance and contextual performance through pressure. In other words, PFP can negatively and positively affect individual job performance. Especially, our results show that the negative indirect effect of PFP on task performance through pressure is significantly smaller than the positive indirect effect of PFP on task performance through intrinsic motivation (contrast estimate = −0.06, 95% CI [−0.07, −0.05]), and similar results are obtained when the outcome variable is contextual performance (contrast estimate = −0.05, 95% CI [−0.06, −0.04]). This suggests that the positive effects of PFP clearly outweigh the negative effects it brings, which to some extent responds to the doubts about the role of PFP in current PFP studies (Deci et al., 1999; Pink, 2009; Frey and Osterloh, 2012).

Fourth, we explored the mechanism of PFP's effect on job performance from an equity perspective and found that different performance aspects have different generative logic. Especially, it appears that the effect of PFP on task performance is primarily driven by distributive justice. These findings were in line with previous research, which stated that distributive justice was more related to personal outcomes (e.g., task performance, job satisfaction) than procedural justice, whereas procedural justice was more related to organizational outcomes (e.g., organizational citizenship behavior, organizational commitment) than distributive justice (McFarlin and Sweeney, 1992; Viswesvaran and Ones, 2002). The most visual feeling that PFP brings to employees is to equate the pay they received with the work effort they invested, and this distributive justice is obvious, so it has a much greater effect on employee task performance than procedural justice has. In other words, even though employees feel that the process is not open and transparent enough or that they cannot participate in the decision-making, they will do everything they can to improve their task performance because they know that PFP will lead to a fair distribution. Furthermore, it appears that the effect of PFP on contextual performance is only driven by procedural justice. In fact, contextual performance can be understood as a kind of reciprocative behavior that does not seek rewards (Organ, 1997). PFP reinforces the perceived procedural justice of employees, which makes them more trusting and committed to the organization, so they will do behaviors that benefit the organization or those around them, rather than doing things that only point to themselves (e.g., enhance in-role effort) (Viswesvaran and Ones, 2002). Previous studies have focused on the effects of PFP on distributive or procedural equity alone (e.g., Chang and Hahn, 2006; Salimäki and Jämsén, 2010), or on the moderating effect of equity on the relationship between PFP and outcomes (e.g., Du and Choi, 2009; Chien et al., 2010; Zhang et al., 2015b), ignoring the mediating effect of perceptions of equity. Our findings not only reveal the “black box” of the process of PFP influencing job performance, but also show the unique effects of different justice mechanisms on different aspects of job performance, which refines the explanatory framework of PFP based on the equity perspective. Therefore, justice-based PFP research may stand as an important complement to the research on the incentive effects of PFP, such as cognitive evaluation-based PFP research.

Fifth, we identified PFP operationalization as one of the important moderators that lead to differential results in the relationship between PFP and its outcomes. The moderator analysis for PFP operationalization indicates that perception measures have stronger relationships with outcomes than proportion measures, besides the PFP-task performance relationship. The key difference between these two measures is that the perception measurement is a subjective approach that focuses more on how employees perceive the pay-performance link, while the proportion measurement is a more objective measure that focuses more on the variable-fixed ratio in actual earnings (Du and Choi, 2009). This subjective measure (i.e., perception measures) considers inter-individual differences in pay expectations and therefore has stronger predictive power than proportion measures. Although the proportion measures are more objective, we recommend that researchers should consider measuring PFP perceptions when assessing PFP in organizational studies, as these PFP measures appear to be superior predictors of PFP outcomes. It is worth noting that our findings show that the proportion measures have stronger relationships with task performance, which is the opposite of what we expected. This result may be because task performance is easier to measure objectively than other outcome variables, and future studies may also explore whether there are other moderators involved.

Finally, we further hypothesized that contextual factors influence the relationship between PFP and outcome variables and found a moderating effect of national culture. Culture affects people's attitudes about the relationship between self and collective (Hofstede et al., 2010). Employees with different degrees of individualistic tendencies may have different opinions when faced with the same compensation system (Fulmer and Shaw, 2018). A collectivist culture is more “in-group” oriented and puts the organization's interests first, which inevitably comes at the sacrifice of individual interests (Chang and Hahn, 2006; Zhang et al., 2021). Therefore, for employees in collectivistic countries, the addition of PFPs that highlight individual values can greatly enhance perceived justice and intrinsic motivation, pay satisfaction, task performance, and contextual performance. However, in an individualistic culture, employees believe that equality, fairness, and autonomy are deserved due to their focus on the “self.” Thus, PFP plays a weaker positive role in individualistic countries than in collectivistic countries. These findings not only help to respond to the conflicting findings of current research on the effects of PFP but also extend the research on moderators of PFP effects.

### Managerial implications

First, companies should pay attention to the incentive role of PFP, especially in designing the appropriate PFP intensity. According to our research findings, PFPs can effectively enhance employees' task performance and contextual performance, pay satisfaction, and other positive attitudes. By designing attractive PFPs, companies can not only improve the job performance of employees within the company, which in turn can improve organizational performance, but also attract more talented people to join the company (Ding et al., 2009). It is important to note that the intensity of PFPs should be suitable (Pokorny, 2008). Our research shows that PFPs can promote intrinsic motivation and thus improve performance on the one hand, but on the other hand, they can also increase employee pressure and undermine employee job performance. Therefore, companies should set a moderate PFP intensity and try to avoid the negative effects caused by too high PFP intensity.

Second, companies should demonstrate fairness in all aspects of compensation allocation, both in terms of outcomes and procedures. PFP can influence employees' job performance by affecting their distributive and procedural justice perceptions. Suppose companies are more concerned about employees' task performance, such as salespeople and production line workers they should focus more on reflecting the fairness of results in pay allocation. Because in our results, the indirect effect of PFP on task performance through distributive justice (0.11, 95% CI [0.10, 0.13]) is nearly six times greater than the indirect effect of PFP on task performance through procedural justice (0.02, 95% CI [0.01, 0.03]). However, companies concerned more about the contextual performance of employees or the job requires more teamwork, such as service industry employees, or R&D employees, are encouraged to focus more on reflecting procedural fairness in pay allocation. As shown in our study, PFP only has an indirect effect on contextual performance through procedural justice (0.07, 95% CI [0.06, 0.09]), and the indirect effect of PFP on contextual performance through distributive justice is not significant (0.01, *ns*). For example, companies could empower employees to participate in decision-making and timely communication about pay allocation (Colquitt et al., 2002).

Third, different national companies should take into account their own national individualistic or collectivistic tendencies to properly utilize the incentive effects of PFP. A more challenging PFP system can be set up for organizations in collectivistic countries. This kind of challenging PFP, which could fully convey the information attribute of PFP, stimulates employees' intrinsic motivation, thus enhancing their performance and work attitude. Management actions can be undertaken for organizations in individualistic countries to enhance employees' attention to collective interests. For example, companies can conduct organizational culture training to align employees with corporate values (Deckop et al., 1999). Companies can also provide organizational support to employees in need, strengthening their collective identity and loyalty. These initiatives help PFP work in individualistic countries on contextual performance, pay satisfaction, etc. In both collectivistic and individualistic countries, managers should create a harmonious atmosphere of support and trust, give employees full autonomy, and weaken the sense of control that PFP causes.

Fourth, companies should fully advocate and communicate to their employees about the PFP they are implementing. If the organizations do not effectively convey the implemented PFP to employees, there will be a significant difference between the employees' perceived PFP and the actual PFP. The information mismatch has the potential to weaken the positive effects of PFP and amplify the negative effects of PFP. According to social information processing theory (Salancik and Pfeffer, 1978), employees' perceptions of PFP are influenced by their surroundings, such as the general organizational environment, immediate leaders, and colleagues (Jiang et al., 2017). Therefore, organizations can enhance employees' positive perceptions of PFP in two ways. Organizations can conduct sessions about PFP to convey the content of PFP to employees directly. Moreover, organizations might also pay attention to line managers' role in compensation allocation. Through the lens of line managers, organizations may improve communication with employees about PFP and increase the transparency of the PFP implementation procedures (Kehoe and Han, 2020).

### Limitations and future research opportunities

First, our meta-analytic study focuses on the effects of PFP on employee-level outcomes. Depending on the level involved, PFP is divided into four main categories: individual PFP, team PFP, organizational PFP, and executive PFP (Gerhart et al., 2009). Although individual PFP is more commonly applied to employees, other levels of PFP may also be co-applied to employees in the workplace, in addition to the executive PFP. Therefore, it would contribute to PFP research and corporate practices if we could distinguish which level of PFP has the greater impact on employees (Garbers and Konradt, 2014). We have also tried to explore the moderating role of PFP levels in our study. Unfortunately, most of the available studies examining the effects of PFP on employee outcomes are individual PFP, and few examine the effects of team PFP on employees outcomes (Rack et al., 2011), as most of the team PFP studies explored the effects of team PFP on the team outcomes. In addition, a small number of extant studies indicated that the sample firms used a mixed PFP (i.e., a combination of two or three types of PFPs from individual PFP, team PFP, and organizational PFP) or simply did not specify the level of PFP in the studies. We made efforts to test the PFP levels as a moderator. Still, there were so few studies (less than or equal to 3) using mixed PFP in each PFP-outcome relationship pair that it was less meaningful to test the moderating effect, and the results showed that the moderating effect of the PFP level was insignificant. Therefore, we did not include this moderating effect test in our study. However, we still believe that the PFP level is a moderator worth exploring and expect future studies to test for this moderating effect when there are sufficient studies in each subgroup.

Second, the number of studies eventually included in the meta-analysis of the PFP-pressure relationship was relatively limited. Although we searched different English and Chinese databases to obtain as many articles as possible, we still did not find more studies that could be included in the meta-analysis. This is due to two reasons: one is that scholars usually use experimental rather than field research studies when studying the effects of PFP on pressure, and the other is that pressure (e.g., negative emotions, fatigue, anxiety) as applied in the studies are not consistent with the definitions in our study (e.g., Levi, 1972; Shirom et al., 1999; Yeh et al., 2009). We presumed that the non-significant moderating effect of both PFP measures and national culture on the relationship between PFP and pressure was also related to the small number of studies that could be included. Future researchers should focus on the role of PFP on pressure and consider additional moderators to weaken the effect of PFP on pressure (Kong et al., 2022), such as organizational support climate, leadership coaching behaviors, individual pressure tolerance, etc.

Third, we explore the mechanisms underlying PFP on job performance from two perspectives and lack integration of these mechanisms. As PFP scholars tend to adopt one lens when explaining the role of PFP, potential synergies between cognitive evaluation-based PFP research and equity-based PFP research remain unknown. Another explanation for the lack of integration efforts is that the two theories focus on different aspects. PFP research based on a cognitive evaluation perspective tends to focus on individuals' fulfillment of intrinsic needs (e.g., competence). PFP research based on an equity perspective tends to focus on an individual's comparison with a referent and will be relatively more rational. Indeed, if we try to combine the correlation matrices in Tables 2 and 4 to test an integrated meditational model, four of the ten equity-cognition cells will be empty. Also, including too many explanatory variables may result in multicollinearity, as in Colquitt et al. (2013). We look forward to future meta-analyses that integrate multiple perspectives into one model to get a more comprehensive understanding of the mechanisms of PFP on job performance. Future research may integrate multiple mechanisms in a single model by combining similar variables. Also, future researchers may open up the “black box” of PFP influencing job performance from more perspectives, such as emotional mechanisms (e.g., positive affect and negative affect) (Schaubroeck et al., 2008), psychological need mechanisms (e.g., perceived autonomy, competence, and relatedness) (Deci and Ryan, 2000).

## Conclusions

In this article, we integrated the empirical studies of PFP conducted in actual work settings to provide a more accurate view of how PFP works in the workplace. Our meta-analysis clearly demonstrates that PFP has a positive effect on job performance in the workplace. To better understand the mechanisms by which PFP affects job performance, our meta-analytic study examined and expanded the theoretical model through two dominant perspectives. For cognitive evaluation, we found that PFP exerted a double-edged sword effect on job performance by increasing employees' intrinsic motivation and pressure. The positive indirect effect of PFP on job performance through intrinsic motivation was slightly greater than the negative indirect effect of PFP on job performance through pressure. For equity, we found that the mediating effect of distributive justice on PFP and task performance was significantly stronger than procedural justice. The relationship between PFP and contextual performance was mediated only by procedural justice. We also found a direct positive effect of PFP on both task performance and contextual performance, which encourages future research to explore more mediating variables. In addition, our findings highlighted the moderating role of national culture and PFP operationalization on the effect of PFP.

## Data availability statement

The original contributions presented in the study are included in the article/supplementary material, further inquiries can be directed to the corresponding author.

## Author contributions

YC and ZZ planned the study and built the study structure framework. YC, ZZ, and JZ wrote the manuscript. YC, JZ, and TY analyzed the data. CL, XZ, and TY coded the data. All authors contributed to the article and approved the submitted version.
